# Quantification of Chlorides and Sulphates on Concrete Surfaces Using Portable X-ray Fluorescence. Optimization of the Measurement Method Using Monte Carlo Simulation

**DOI:** 10.3390/ma14247892

**Published:** 2021-12-20

**Authors:** Servando Chinchón-Payá, Julio E. Torres Martín, Antonio Silva Toledo, Javier Sánchez Montero

**Affiliations:** Instituto Eduardo Torroja Ciencias de la Construcción (IETcc-CSIC), Calle de Serrano Galvache, 4, 28033 Madrid, Spain; juliotorres@ietcc.csic.es (J.E.T.M.); a.silva@ietcc.csic.es (A.S.T.); javier.sanchez@csic.es (J.S.M.)

**Keywords:** concrete, chlorides, sulphates, pXRF, Monte Carlo simulation

## Abstract

A correct assessment of the pathologies that can affect a reinforced concrete structure is required in order to define the repair procedure. This work addresses the challenge of quantifying chlorides and sulphates directly on the surface of concrete. The quantification was carried out by means of X-ray fluorescence analysis on the surface of concrete specimens at different points with portable equipment. Concrete prisms were made with different amounts of NaCl and Na_2_SO_4_. To avoid the influence of coarse aggregate, a qualitative estimate of the amount of coarse aggregate analyzed has been made, although the results show that there is no significant influence. Monte Carlo simulations were carried out in order to establish the necessary number of random analyses of the mean value to be within an acceptable range of error. In the case of quantifying sulphates, it is necessary to carry out six random analyses on the surface, and eight measurements in the case of quantifying chlorides; in this way, it is ensured that errors are below 10% in 95% of the cases. The results of the study highlight that a portable XRF device can be used in situ to obtain concentrations of chlorides and sulphates of a concrete surface with good accuracy. There is no need to take samples and bring them to a laboratory, allowing lower overall costs in inspection and reparation works.

## 1. Introduction

The sulphates present in mortars and concretes may come from the gypsum or anhydrite added to the clinker in the cement manufacturing process as a setting retarder, or they may have been incorporated once cement has hydrated and hardened. An excess of sulphates can be harmful, since if they react with hydrated calcium aluminates, secondary ettringite is formed, while they react with CSH gel, thaumasite can be formed [1,2,3]. The appearance of secondary ettringite causes tensions inside the concrete due to the increase in volume [4], while the formation of thaumasite leads to the disappearance of the CSH gel, which is the main cohesive compound in cement.

However, the sulphates present in mortar or concrete may not be part of these degradation products; rather, they may be adsorbed on other solid compounds or be part of the pore solution. These sulphates will not be harmful if they are not mobilized and react with the compounds mentioned above. There are also cements that, due to their lower calcium aluminate content and/or the presence of substituent additions, are more resistant to sulphates [5].

For all these reasons, there are no sulphate concentration thresholds from which it can be deduced that if these values are exceeded, there is a sulphate attack. Quantifying these ions should be taken merely as an indication and should be complemented by a broader study. Other types of tests on the mortar or concrete are necessary to be able to conclude the existence of an attack, usually scanning electron microscopy (SEM) and X-ray diffraction (XRD) [6,7] together with the Rietveld method to quantify crystalline compounds [8,9].

On the other hand, chloride ions are one of the main causes of deterioration of reinforced concrete structures, causing corrosion of the reinforcement. Chloride causes the breakage of the passive layer that protects the steels, exposing them to further oxidation [10,11,12]. Corrosion of reinforcement can lead to cracks in the concrete and brittle failure of structural members [13,14,15,16,17,18,19,20,21].

Although there is no agreement on a limit of chloride concentration in concrete that causes corrosion of reinforcement [10,22], international standards establish different limits. For example, in the case of the Spanish Structural Concrete Instruction, EHE-08, the limit is set at 0.6% with respect to the weight of cement.

There are different methods of sulphate quantification. Standard methods involve dissolving the solid sample and precipitating the sulphates using a barium salt and then gravimetrically quantifying the initial sulphates in the sample. This methodology requires an adequate chemical analysis laboratory and the analysis is tedious and time consuming. Its reliability or reproducibility is acceptable [23].

The reference method for the quantification of chlorides is based on the Volhard method, by which the chlorides present in a solution are precipitated by the addition of a silver salt to form an insoluble silver chloride [24]. The detection of the end point of this titration can be carried out in different ways, the most usual being the use of colorimetric indicators or by potentiometric methods [23,25]. As in the case of sulphates, the quantification of chlorides in concrete requires a first dissolution or digestion process of the sample, by which all these ions pass from the solid to the liquid phase.

X-ray fluorescence (XRF) is a spectroscopic technique for elemental quantification. Fluorescence is a physical process that occurs in response to radiation by high-energy beams at a solid sample and occurs as a consequence of electronic relaxation when such radiation disappears. The response is element specific and proportional to its concentration in the sample. This technique can be used to calculate the sulphur concentration expressed as SO_3_ in a solid sample, but it is also capable of quantifying the specific signal of the chlorides present in a cementitious matrix. It should be noted that, as stated in the standards, all quantification methods other than those mentioned above as standardized require prior calibration [23].

Handheld X-ray fluorescence equipment (pXRF) allow this technique to be used for elemental analysis in works, buildings, facilities, and infrastructures far from the laboratory, since the analyzer is a small and lightweight device [26]. On the other hand, the portable equipment has a lower accuracy than standard instruments.

Handheld X-ray equipment (pXRF) generally comes with an internal calibration that allows the quantification of elements in a semi-quantitative way, but as in any instrumental technique, it is advisable to calibrate the equipment to determine the desired elements in the matrix in which they are found.

During the inspection of a work, installation, or infrastructure, it is very useful to know at the moment the cause of any apparent degradation of concrete. Normally, when it is necessary to know the elemental composition of the concrete, samples have to be taken and sent to a specialized laboratory for analysis. When the objective is even more specific, such as precisely delimiting the most affected areas in order to define and repair with greater objectivity, the increase in the time required for the entire process significantly increases the costs of these repair stages.

The purpose of this study is to use pXRF equipment to directly quantify the concentration of chlorides and sulphates on the surface of concrete. Given the large number of variables that can influence this concentration, a previous experimental campaign has been carried out in which several concrete specimens with known concentrations of these ions have been analyzed in the laboratory. A large number of punctual analyses have been carried out in order to know whether their average value is close to the real value. Subsequently, by means of a Monte Carlo simulation, the number of punctual analyses necessary for their mean value to be as close as possible to the real value within an acceptable error has been obtained.

Monte Carlo simulation is a statistical analytical method used to mathematically model real systems and estimate the probability of obtaining the expected outcome [27,28]. Since concrete is considered a heterogeneous material in its elemental composition, it can be modeled or simulated using the Monte Carlo method. It is used in a wide range of disciplines, from economics to engineering [29,30,31,32,33], and involves the use of random numbers to calculate a response of the modeled system. Although it can be carried out in multiple ways, Monte Carlo simulation has certain similarities for each case study:

The pseudo-population or model that represents the true population of interest is determined.

Samples are taken from this pseudo-population.

A value of the statistic parameter of interest is calculated and stored.

Repeat steps 2 and 3 for *N* trials.

Finally, the values of *N* found in Step 4 are used to study the distribution of the statistic parameter.

## 2. Materials and Methods

**Figure 1:**
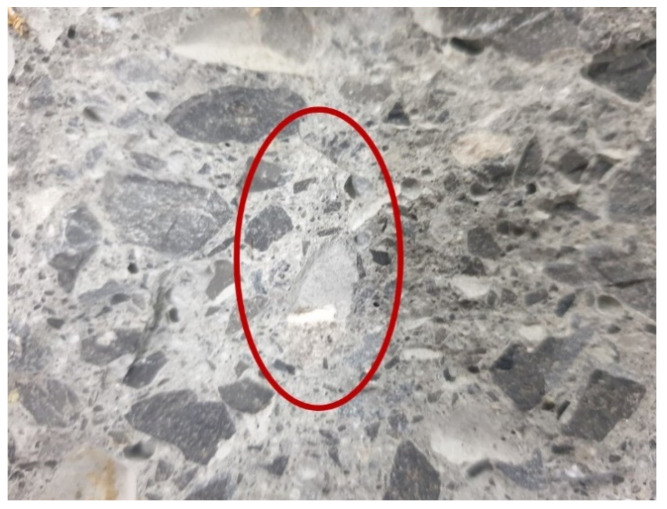
Sample image and analysis spot (in red).

The computer software for managing the pXRF shows the spot or analysis region where the analysis is carried out (see Figure 1). The photograph shows the surface of concrete with cement paste and aggregates of different grain size. It is to be expected that the quantitative result of the elements incorporated in the cement paste will be influenced by the amount of aggregate in the analysis spot. Therefore, in order to know the concentration of chlorides and sulphates on the concrete surface, numerous analyses were carried out, scoring each of them with a factor from 0 to 4 depending on whether the analysis was carried out on areas with less (0%) or more (100%) coarse aggregate.

The graphical representation of the concentrations obtained as a function of the factor allows knowledge of the influence of the coarse aggregate in the quantification by this technique, since the main sources of sulphates and chlorides are found in the cementitious matrix.

Once the influence of the aggregate was verified, a simulation was carried out using the Monte Carlo method to determine the number of random point analyses that achieve an average value as close as possible to the real value. This simulation followed the following process:

- Identification of the optimal distribution of sulphate and chloride quantification results. Once the distribution is defined, the descriptive statistic parameters for each one of them are defined.
- In the simulation itself, random values are taken for each of the distributions defined by the previous statistic parameters. The number of random values is increased so that when computing the averages, it is possible to identify which average is close to the real value with an error of 10%.

### 2.1. Materials

Concrete specimens of dimensions 15 cm × 15 cm × 15 cm were made. A known percentage of Na_2_SO_4_ or NaCl (dissolved in the mixing water) was introduced into each of the specimens.

The cement used was a Type I cement and the water/cement ratio was 0.45. The concrete specimens were dosed considering a density of 2400 Kg/m^3^ and a cement quantity of 350 Kg/m^3^ in all cases (see Table 1).

In the case of specimens with known chloride content, they had a final chloride concentration of 0%, 0.18%, 0.37%, and 0.74% with respect to the total weight of the concrete. In the case of sulphate quantification, concrete specimens had added sulphate percentages of 0%, 0.25%, 0.5%, and 1% with respect to the total weight of the concrete. These percentages represent a wide range that allows checking the reliability of the measurement and, transformed into weight of cement, represent 0%, 1.36%, 2.72%, and 5.44% of added chlorides, and 0%, 1.8%, 3.7%, and 7.4% of added sulphates, respectively.

The specific quantities for the preparation of each of the specimens are shown in Table 1 and Table 2.

**Figure 2:**
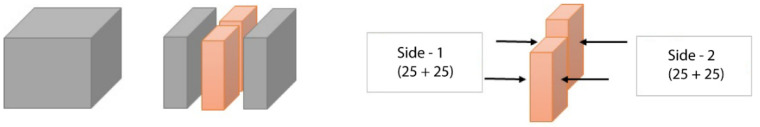
Concrete specimen and breakage scheme with sections for pXRF analysis.

Once the specimens were demolded and cured during 28 days in a humid chamber, they were split transversely into three pieces to obtain more surfaces for analysis (see Figure 2). In order to do so, before applying increasing pressure in the vertical plane, notches were made to obtain fracture surfaces as straight as possible. Cutting with water was avoided to prevent contamination or washing of the sample surface. Each of the three previous pieces was divided in two to be able to introduce and move each of them inside the workstation. Once the surfaces were prepared, 50 points per fracture surface were analyzed, corresponding to a total of 200 points per specimen, using handheld XRF equipment (pXRF).

### 2.2. Analysis

The portable XRF equipment used was an Olympus Innov-X, Delta model. Of the available working modes (soils and geochemistry), the soils mode was selected. The quantification results are expressed in parts per million (ppm). Chlorides are quantified directly as chloride ions, but sulphate quantification is expressed as total sulphur (S) and a conversion to SO_4_^2−^ or SO_3_ is necessary.

A workstation from the same manufacturer was also available to connect the pXRF equipment and analyze the samples without the analyst being subjected to radiation or having to hold the equipment manually during the analysis. The workstation was connected to a computer to monitor the tests from the computer. Thanks to the fact that the analysis interface of the equipment’s software allows us to observe the area being analyzed, we can easily detect whether we are in an area with more or less aggregate. Each analysis was categorized from 0 to 4 according to the amount of aggregate, with 0 being all paste (and fine aggregate) and 4 being an analysis performed on 100% coarse aggregate.

Although the pXRF equipment has an internal calibration, prior to the concrete surface analyses, samples with different amounts of sulphates and chlorides were prepared to calibrate perfectly the response of the instrument to samples whose matrix was similar to the object of analysis. The results of this calibration with powder samples of test concretes [26] indicate the need to apply two correction factors in the quantification of chlorides (1.16) and sulphates (0.85).

### 2.3. Simulation Using Monte Carlo Method

In practice, in order to obtain the average concentration values in concrete, it is not feasible to carry out two hundred measurements on a surface; a Monte Carlo simulation has been carried out with the data obtained in the experimental stage. Starting from the statistical data (the mean of the concentration values per specimen and its standard deviation) and the distribution to which the experimental data fit, it is possible to obtain the optimum number of analyses to be performed on a concrete surface to be within an acceptable error with minimum uncertainty. The process follows the scheme below:

1. Fit the experimental data to an appropriate distribution and obtain the characteristic values, such as averages (m_x_) and standard deviation (sd_x_). Distributions will be obtained for each analyzed element and its concentration in the concrete.
2. Simulation of a single surface analysis starting from the above distribution with the statistical parameters m_x%_ and sd_x%_. One million random analyses were simulated for each element and concentration.
3. The previous point is repeated for the case of performing 2, 3, 4, …, 20 analyses on the same surface.
4. The corresponding means and deviations of the previous simulations are calculated and plotted together with confidence intervals of ±95% and ±90%, as well as the lines corresponding to ±10% error of the average.

## 3. Results and Discussion

### 3.1. Sulphate Quantification

**Figure 3:**
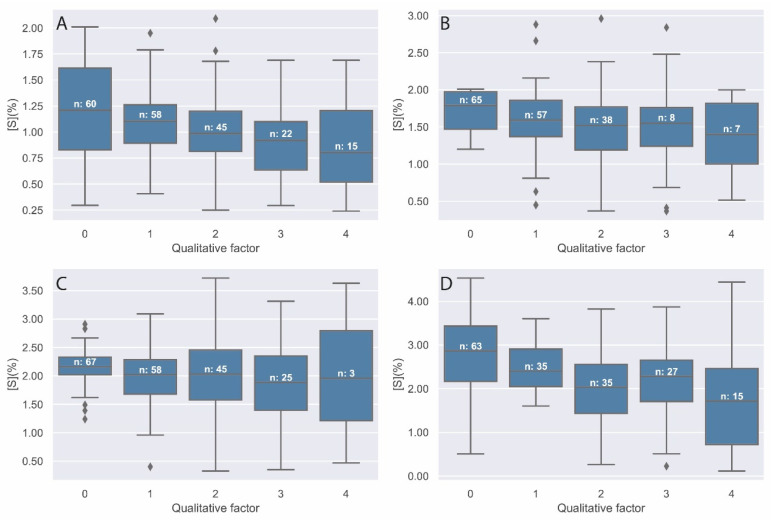
Representation of sulphur concentration (%) in specimens (**A**) S1, (**B**) S2, (**C**) S3, and (**D**) S4 distributed by coarse aggregate quality factor (0–4).

Figure 3 shows the results of sulphate quantification on the surface of the different specimens as a function of the occupancy factor, or amount of aggregate observed at the time of analysis. Figure 3A corresponds to a SO_4_ concentration of 0%, Figure 3B to 0.25%, Figure 3C to 0.5%, and Figure 3D to 1%. Each analysis has been ranked from 0 to 4 according to the amount of aggregate included in the analysis.

As can be seen in the box and whisker plots in Figure 3, although the dispersion of the results is large, the sulphates present similar average values within each of the dosages. In other words, the amount of aggregate present at the time of analysis does not significantly influence the analysis.

### 3.2. Chloride Determination

**Figure 4:**
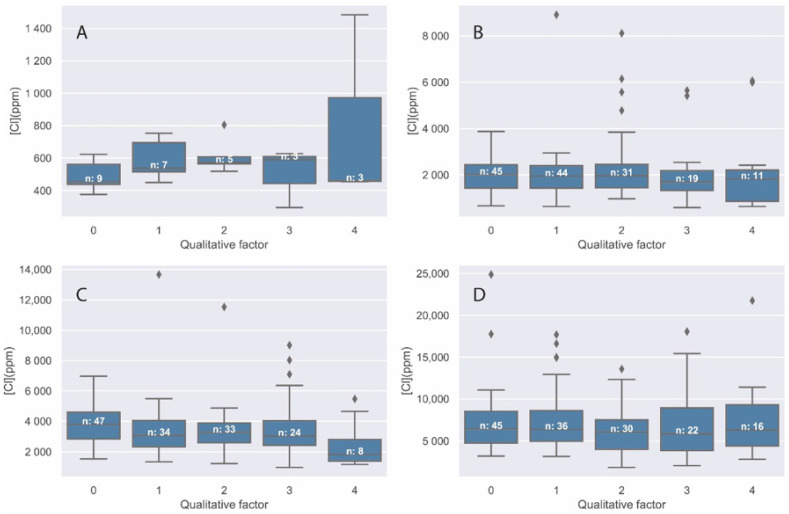
Cl quantification in ppm for (**A**) Cl1, (**B**) Cl2, (**C**) Cl3, and (**D**) Cl4 specimens distributed by coarse aggregate qualitative factor (0–4).

The following box-and-whisker plots show the chloride concentration as a function of the aggregate occupancy factor. It was decided not to omit the outliers; of which it should be noted that they are always on the high concentration side. This could be due to a higher point concentration on the specific surface of analysis or to recrystallization by evaporation in concrete pores. Figure 4A corresponds to a chloride concentration of 0%, Figure 4B to 0.18%, Figure 4C to 0.37%, and Figure 4D to 0.74%.

The number of observations for the 0% chloride specimen is significantly lower than for the rest of the specimens, since a large number of spot analyses gave quantification results below the detection limit of the technique.

In view of the results in Figure 4, it can be inferred that the mean values of chloride concentration analyzed in each of the specimens are independent of the amount of aggregate within the analyzed zone. A large dispersion of the results within each factor is also observed, with values in a very wide range both above and below the average values.

### 3.3. Simulation by Monte Carlo Method

In order to perform an optimal simulation of the sample, it is necessary to know the distribution of the sample and to use the representative statistics of this distribution. The histograms of each of the samples are shown graphically below, together with the representation of the normal and lognormal density functions for each of them. These representations show which distribution best fits each of the histograms represented.

**Figure 5:**
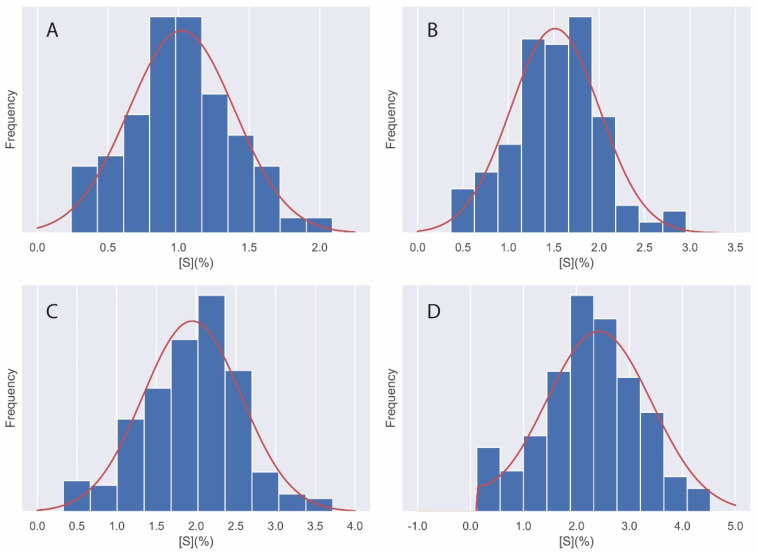
Histogram distributions for (**A**) S1, (**B**) S2, (**C**) S3, and (**D**) S4. The normal probability density (red line) has also been added.

Figure 5 shows the histograms of sulphate quantification in the different specimens. For each of these distributions the normal density functions have been added. Actually, for the case of D4, the folded normal distribution is shown instead of the normal one because the normal representation for this case reached negative values, a fact that does not make physical sense since there cannot be negative sulphate concentrations in concrete.

The statistic parameters that represent a normal distribution are the average and the standard deviation. In the case of the sulphate quantification experiments, these are shown in Table 3. The percentage of sulphates corresponds to the total of the sample.

**Figure 6:**
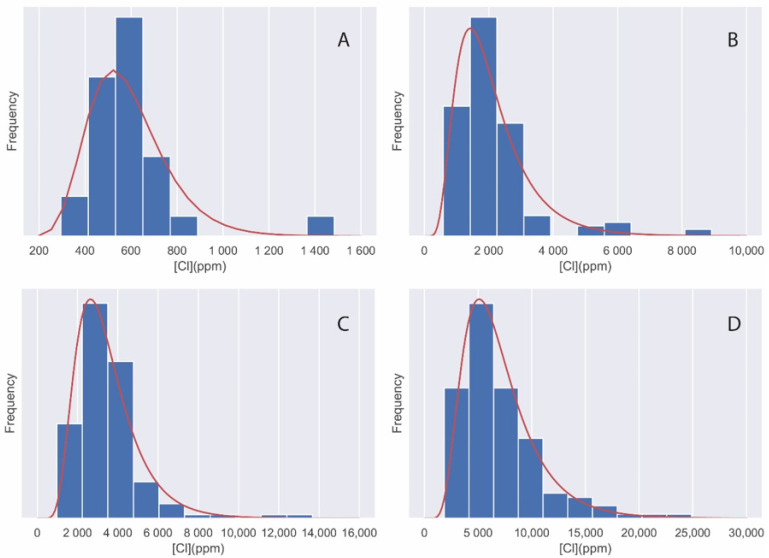
Histogram distributions for (**A**) Cl1, (**B**) Cl2, (**C**) Cl3, and (**D**) Cl4. The lognormal probability density (red line) has also been added.

Histograms of the chloride quantification results of the different test specimens are shown in Figure 6. In contrast to the case of sulphates, the lognormal density functions are also shown as fits to the concentration distributions.

Figure 6 shows that, for the case of the quantification of chlorides on the surface of concrete, the best fitting distribution is the lognormal distribution. For this type of distribution, the descriptive statistics are meanlog and sdlog. These are shown in Table 4.

Random values within the distributions found were then sampled. These random values correspond to the number of point analyses on the concrete surface. For the Monte Carlo simulation, the following process was followed:

1. A random value is taken that meets the conditions for each of the distributions; that is, they are within a normal distribution with the mean and standard deviation of Table 3 for sulphates (folded normal for S4) and within a lognormal distribution with meanlog and sdlog indicated in Table 4 for chlorides. The process is replicated one million times.
2. Next, we simulate taking two values with the same conditions as in Step 1 and calculate the mean and standard deviation. The process is replicated one million times and the mean and standard deviation values (mean_2_ and sd_2_) are saved.
3. The same process is simulated for 3, 4, 5, 6, … up to 10 values. For each one, it is replicated one million times and the mean and standard deviation values are saved (mean_3_ and sd_3_, mean_4_ and sd_4_, mean_5_ and sd_5_, … up to mean_10_ and sd_10_).
4. Finally, the mean values are plotted together with the confidence intervals and a 10% error. That is, the values mean_n_, 95% IC, 90% IC, error +10%, and error −10% calculated are plotted according to:
  SE = sd_n_/Sqr(n)
  IC 95% = mean_n_ + 1.96 * SE
  IC 90% = mean_n_ + 1.645 * SE
  Error ±10% = mean_n_ ± 0.1 * mean_n_

To check the number of point analyses on a concrete surface to be carried out with the portable X-ray equipment, it is sufficient to check the value of x at the intersection of the 95% IC (or 90% IC) curves with the 10% error on the graphs obtained.

**Figure 7:**
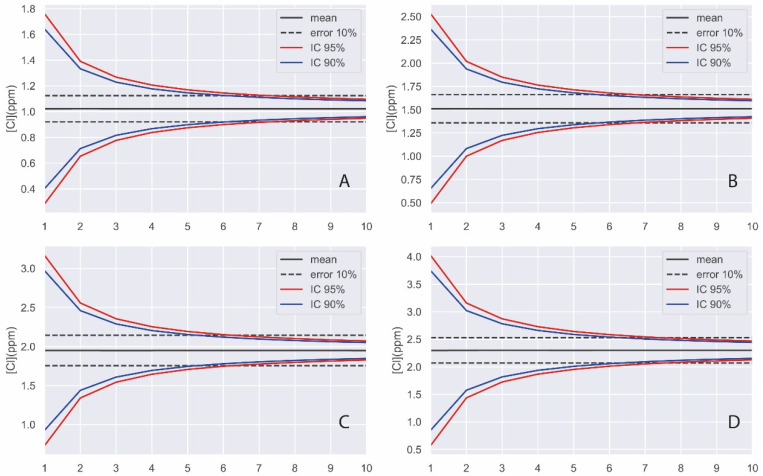
Monte Carlo simulation results of the normal distributions of sulphate concentration on the surface of concrete specimens (**A**) S1, (**B**) S2, (**C**) S3, and (**D**) S4.

The results of the Monte Carlo simulation of sulphate quantification on the surfaces of the different concrete specimens are shown in Figure 7.

As can be seen in Figure 7, with a random number of total analyses of six on the concrete surface, we ensure that the mean value is, 95% of the time, within the defined error of 10%.

**Figure 8:**
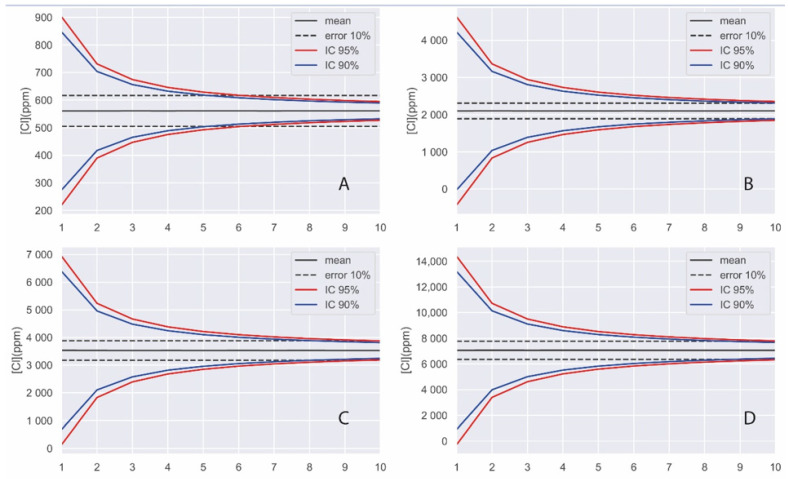
Monte Carlo simulation results of the lognormal distributions of chloride concentration on the surface of concrete specimens (**A**) Cl1, (**B**) Cl2, (**C**) Cl3, and (**D**) Cl4.

The results of the simulation for the case of chloride quantification are shown in Figure 8.

In view of the simulation results, it can be concluded that with about eight random measurements (without considering whether we are analyzing more or less aggregate) on the surface of a concrete, we are quantifying chlorides within a ±10% error with a confidence interval of 95%, or in other words, in only 5% of the cases we cannot be sure of having an error of less than 10%.

## 4. Conclusions

The study presents the results of an experiment in the quantification of chlorides and sulphates in concrete using handheld X-ray fluorescence equipment (pXRF). Calibration for quantification directly on the surface is shown, without the need for sample extraction or sample preparation.

It has been observed that the concentration of these ions does not depend on the amount of coarse aggregate analyzed, since the dispersion around the mean value is much greater than the effect of the aggregate itself. The distribution of the quantification results for sulphate conforms to a normal density function, while chlorides conform to a lognormal one.

A simulation was carried out using the Monte Carlo method based on the mean and standard deviation statistics of the sulphate concentrations in each of the specimens and the medianlog and sdlog values in the case of chlorides. The Monte Carlo method allows knowledge of what mean of *n* values is within the 10% error in 95% and 90% of the occasions, with being *n* the number of spot analyses to be done with the pXRF device on the surface of the concrete.

The results show that by performing nine analyses on the surface of the concrete, the chloride content can be determined with an error of less than 10% at a 95% confidence interval. In relation to sulphates, the results of the study indicate that with a number of six analyses, we can obtain its content with an error of less than 10% at a 95% confidence interval.

Although the quantification analyses obtained using the pXRF equipment are not as accurate as traditional X-ray fluorescence, the results are acceptable if we take into account the necessary prior calibration for each piece of equipment and its use in cement-based materials.

## Figures and Tables

**Table 1:** Dosage of the concrete specimens for the quantification of sulphates and chlorides.

Aggregate (6919 g.).			Cement (g.) *	Water (g.) **
0/4	4/10	10/20	1181	591
2768	2975	1176		

* Total weight of cement and Na_2_SO_4_ and/or NaCl additions. ** 24.81 g of superplasticizer was also added to the mixing water.

**Table 2:** Quantities of Na_2_SO_4_ and NaCl added to each specimen. The corresponding percentages relative to cement and concrete are shown.

Name	Weight Na_2_SO_4_ (g.)	%SO_4_ Cement	%SO_4_ Concrete	Weight NaCl (g.)	%Cl Cement	%Cl Concrete
**S1**	0.00	0.00	0.00	-	-	-
**S2**	32.22	1.84	0.25	-	-	-
**S3**	64.45	3.69	0.50	-	-	-
**S4**	128.89	7.38	1.00	-	-	-
**Cl1**	-	-	-	0.00	0.00	0.00
**Cl2**	-	-	-	26.48	1.36	0.18
**Cl3**	-	-	-	52.93	2.72	0.37
**Cl4**	-	-	-	105.87	5.44	0.74

**Table 3:** Statistics used for Monte Carlo simulation. Average values and standard deviation for specimens S1, S2, S3, and S4. Data without applying the correction factor directly obtained from the pXRF equipment.

Name	Mean (%)	SD
**S1**	1.02	0.38
**S2**	1.51	0.49
**S3**	1.95	0.62
**S4**	2.26	0.91

**Table 4:** Descriptive statistics of the lognormal distribution (meanlog and sdlog) for the results of chloride quantification in the different specimens.

Name	Meanlog (%)	SDlog
**Cl1**	6.34	0.28
**Cl2**	7.52	0.51
**Cl3**	8.06	0.43
**Cl4**	8.75	0.46

## Data Availability

The data presented in this study are available on request from the corresponding author. The data are not publicly available due to they belong to RETINEO.

## References

[1] Hooton R.D. (2013). A Review of Different Forms of Sulfate Attack.

[2] Neville A. (2004). The confused world of sulfate attack on concrete. Cem. Concr. Res..

[3] Chinchón-Payá S., Aguado A., Nugteren H.W., Chinchón S. (2015). External sulfate attack in dam concretes with thaumasite formation. Mater. Constr..

[4] Sarkar P.K., Mitra N., Prasad D. (2019). Molecular level deformation mechanism of ettringite. Cem. Concr. Res..

[5] Chinchón-Payá S., Torres J., Rebolledo N., Sánchez J. (2021). Evaluación del estado de elementos estructurales del Mercado de Legazpi: Ataque por sulfatos al hormigón y corrosión de las armaduras. Inf. Constr..

[6] İnan Sezer G., Ramyar K., Karasu B., Burak Göktepe A., Sezer A. (2008). Image analysis of sulfate attack on hardened cement paste. Mater. Des..

[7] Chinchón-Payá S. (2013). Áridos Reactivos en Hormigones de Presa. Reacción Sulfática con Formación de Thaumasita. Ph.D. Thesis.

[8] Young R.A. (1995). The Rietveld Method.

[9] Rodríguez-Carvajal J. FULLPROF: A Program for Rietveld Refinement and Pattern Matching Analysis. Proceedings of the Satellite Meeting on Powder Diffraction of the XV Congress of the IUCr.

[10] Angst U.M., Geiker M.R., Alonso M.C., Polder R., Isgor O.B., Elsener B., Wong H., Michel A., Hornbostel K., Gehlen C. (2019). The effect of the steel–concrete interface on chloride-induced corrosion initiation in concrete: A critical review by RILEM TC 262-SCI. Mater. Struct..

[11] Pachón-Montaño A., Sánchez-Montero J., Andrade C., Fullea J., Moreno E., Matres V. (2018). Threshold concentration of chlorides in concrete for stainless steel reinforcement: Classic austenitic and new duplex stainless steel. Constr. Build. Mater..

[12] Markeset G. (2009). Critical chloride content and its influence on service life predictions. Mater. Corros..

[13] Sanchez J., Fullea J., Andrade C. (2017). Fracto-surface mobility mechanism in high-strength steel wires. Eng. Fract. Mech..

[14] Sanchez J., Fullea J., Andrade C. (2017). Corrosion-induced brittle failure in reinforcing steel. Theor. Appl. Fract. Mech..

[15] Morales J.A., Torres J., Rebolledo N., Sánchez J. (2019). Experimental and Statistical Analysis of the Corrosion in Tendons in Contact With Water. Front. Mater..

[16] Iordachescu M., Valiente A., Pérez-Guerrero M., Elices M. (2018). Environment-assisted failure of structural tendons for construction and building applications. Constr. Build. Mater..

[17] Keßler S., Fischer J., Straub D., Gehlen C. (2014). Updating of service-life prediction of reinforced concrete structures with potential mapping. Cem. Concr. Compos..

[18] Belletti B., Rodríguez J., Andrade C., Franceschini L., Sánchez Montero J., Vecchi F. (2020). Experimental tests on shear capacity of naturally corroded prestressed beams. Struct. Concr..

[19] Vecchi F., Belletti B., Franceschini L., Andrade C., Rodriguez J., Montero S.J. Flexural Tests on Prestressed Beams Exposed to Natural Chloride Action. Proceedings of the FIB CACRCS DAYS 2020.

[20] Belletti B., Vecchi F., Bandini C., Andrade C., Montero J.S. (2020). Numerical evaluation of the corrosion effects in prestressed concrete beams without shear reinforcement. Struct. Concr..

[21] Possan E., Dal Molin D.C.C., Andrade J.J.O. (2018). A conceptual framework for service life prediction of reinforced concrete structures. J. Build. Pathol. Rehabil..

[22] Angst U., Elsener B., Larsen C.K., Vennesland Ø. (2009). Critical chloride content in reinforced concrete—A review. Cem. Concr. Res..

[23] EUROPEAN STANDARD UNE-EN 196-2:2014 (2014). Métodos de Ensayo de Cementos. Parte 2: Análisis Químico de Cementos.

[24] Sui S., Wilson W., Georget F., Maraghechi H., Kazemi-Kamyab H., Sun W., Scrivener K. (2019). Quantification methods for chloride binding in Portland cement and limestone systems. Cem. Concr. Res..

[25] EUROPEAN STANDARD UNE-EN 14629:2007 (2007). Productos y Sistemas para la Protección y Reparación de Estructuras de Hormigón. Métodos de Ensayo. Determinación del Contenido en Cloruros en el Hormigón Endurecido.

[26] Chinchón-Payá S., Torres Martín J.E., Ramos N.R., Montero J.S. (2021). Use of a handheld x-ray fluorescence analyser to quantify chloride ions in situ: A case study of structural repair. Materials.

[27] Kalos M.H., Whitlock P.A. (2008). Monte Carlo Methods.

[28] Robert C., Casella G., Statistics S.T. (2004). Monte Carlo Statistical Methods.

[29] Bojórquez J., Ponce S., Ruiz S.E., Bojórquez E., Reyes-Salazar A., Barraza M., Chávez R., Valenzuela F., Leyva H., Baca V. (2021). Structural reliability of reinforced concrete buildings under earthquakes and corrosion effects. Eng. Struct..

[30] Alwash M., Breysse D., Sbartaï Z.M. (2017). Using Monte-Carlo simulations to evaluate the efficiency of different strategies for nondestructive assessment of concrete strength. Mater. Struct. Constr..

[31] Wang X.F., Yang Z.J., Yates J.R., Jivkov A.P., Zhang C. (2015). Monte Carlo simulations of mesoscale fracture modelling of concrete with random aggregates and pores. Constr. Build. Mater..

[32] Huang Y.J., Yang Z.J., Chen X.W., Liu G.H. (2016). Monte Carlo simulations of meso-scale dynamic compressive behavior of concrete based on X-ray computed tomography images. Int. J. Impact Eng..

[33] Possan E., De Oliveira Andrade J.J. (2014). Markov chains and reliability analysis for reinforced concrete structure service life. Mater. Res..

